# Mismatch repair deficiency, chemotherapy and survival for resectable gastric cancer: an observational study from the German staR cohort and a meta-analysis

**DOI:** 10.1007/s00432-022-03953-y

**Published:** 2022-02-25

**Authors:** T. Stolze, S. Franke, J. Haybaeck, M. Moehler, P. P. Grimminger, H. Lang, W. Roth, I. Gockel, N. Kreuser, H. Bläker, C. Wittekind, F. Lordick, M. Vieth, L. Veits, O. Waidmann, P. Lingohr, U. Peitz, C. Schildberg, M. Kruschewski, N. Vassos, E. Goni, C. J. Bruns, K. Ridwelski, S. Wolff, H. Lippert, J. Schumacher, P. Malfertheiner, M. Venerito

**Affiliations:** 1 Department of Gastroenterology, Hepatology and Infectious Diseases, Otto-von-Guericke University Hospital Magdeburg, Magdeburg, Germany; 2 Institute of Pathology, Otto-von-Guericke University Hospital Magdeburg, Magdeburg, Germany; 3 Institute of Pathology, Neuropathology and Molecular Pathology, Medical University of Innsbruck, Innsbruck, Austria; 4 Diagnostic and Research Center for Molecular BioMedicine, Institute of Pathology, Medical University Graz, Graz, Austria; 5 Department of Internal Medicine I, Johannes Gutenberg-University of Mainz, Mainz, Germany; 6 Department of General, Visceral and Transplant Surgery, Johannes Gutenberg-University of Mainz, Mainz, Germany; 7 Institute of Pathology, University Hospital Mainz, Mainz, Germany; 8 Department of Medicine II and University Cancer Center Leipzig (UCCL), Leipzig University Medical Center, Leipzig, Germany; 9 Institute of Pathology, University Hospital Leipzig, Leipzig, Germany; 10 University Cancer Center Leipzig, University Hospital Leipzig, Leipzig, Germany; 11 Institute of Pathology, Friedrich-Alexander University Erlangen-Nuremberg, Klinikum Bayreuth, Bayreuth, Germany; 12 Department of Internal Medicine 1, Main Area Gastroenterology and Hepatology, University Hospital Frankfurt, Frankfurt am Main, Germany; 13 University Cancer Center, University Hospital Frankfurt, Frankfurt am Main, Germany; 14 Department of General, Visceral, Thoracic and Vascular Surgery, University Hospital Bonn, Bonn, Germany; 15 Department of Gastroenterology, Raphaelshospital, Münster, Germany; 16 Department of General and Visceral Surgery, Brandenburg, University Hospital of Visceral Surgery, Brandenburg, Germany; 17 Department of General and Visceral Surgery, Hospital Frankfurt (Oder), Frankfurt (Oder), Germany; 18 Division of Surgical Oncology and Thoracic Surgery, Department of Surgery, University Medical Center Mannheim, University of Heidelberg, Mannheim, Germany; 19 Department of Medicine II, University Hospital, LMU Munich, Munich, Germany; 20 Department of General, Tumor and Transplantation Surgery, University Hospital Cologne, Köln, Germany; 21 Department of General and Visceral Surgery, Municipal Hospital, Magdeburg, Germany; 22 AN-Institute of Quality Assurance in Operative Medicine, Otto-von-Guericke University Hospital, Magdeburg, Germany; 23 Department of General, Visceral, Vascular and Transplantation Surgery, Otto-von-Guericke University Hospital, Magdeburg, Germany; 24 Human Genetics Center, Philipps University of Marburg, Marburg, Germany; 25 Department of Gastroenterology, Hepatology and Infectious Diseases, Medizinische Fakultät der Otto-Von-Guericke-Universität, Leipziger Straße 66, 39120 Magdeburg, Germany

**Keywords:** Gastric cancer, Mismatch repair deficiency, Chemotherapy, Survival, Meta-analysis

## Abstract

**Purpose:**

In a post hoc analysis of the MAGIC trial, patients with curatively resected gastric cancer (GC) and mismatch repair (MMR) deficiency (MMRd) had better median overall survival (OS) when treated with surgery alone but worse median OS when treated with additional chemotherapy. Further data are required to corroborate these findings.

**Methods:**

Between April 2013 and December 2018, 458 patients with curatively resected GC, including cancers of the esophagogastric junction Siewert type II and III, were identified in the German centers of the staR consortium. Tumor sections were assessed for expression of MLH1, MSH2, MSH6 and PMS2 by immunohistochemistry. The association between MMR status and survival was assessed. Similar studies published up to January 2021 were then identified in a MEDLINE search for a meta-analysis.

**Results:**

MMR-status and survival data were available for 223 patients (median age 66 years, 62.8% male), 23 patients were MMRd (10.3%). After matching for baseline clinical characteristics, median OS was not reached in any subgroup. Compared to perioperative chemotherapy, patients receiving surgery alone with MMRd and MMRp had a HR of 0.67 (95% CI 0.13–3.37, *P* = 0.63) and 1.44 (95% CI 0.66–3.13, *P* = 0.36), respectively. The meta-analysis included pooled data from 385 patients. Compared to perioperative chemotherapy, patients receiving surgery alone with MMRd had an improved OS with a HR of 0.36 (95% CI 0.14–0.91, *P* = 0.03), whereas those with MMRp had a HR of 1.18 (95% CI 0.89–1.58, *P = 0.26*).

**Conclusion:**

Our data support a positive prognostic effect for MMRd in GC patients treated with surgery only and a differentially negative prognostic effect in patients treated with perioperative chemotherapy. MMR status determined by preoperative biopsies may be used as a predictive biomarker to select patients for perioperative chemotherapy in curatively resectable GC.

## Introduction

Gastric cancer (GC) is the fifth most frequently diagnosed cancer and third leading cause of cancer death globally (Bray et al. 2018). Risk factors that increase the risk of GC include *Helicobacter pylori* gastritis, autoimmune gastritis, age, male gender, smoking, a diet high in salty and smoked foods / low in fruits and vegetables, a family history of GC and a hereditary disposition (Forman and Burley 2006; Venerito et al. 2016; Weise et al. 2020). Furthermore, obesity, gastroesophageal reflux disease and a medium or high socio-economic status all increase the risk for cardia GC (Franck et al. 2021).

The majority of GC are adenocarcinomas, which can be subdivided histologically into intestinal and diffuse types according to the Laurén classification (Laurén 1965). More recently, a molecular classification has been proposed, dividing GC in four subtypes: tumors positive for Epstein-Barr virus, genomically stable (GS), chromosomally unstable (CIN) and microsatellite instable (MSI) tumors (Bass et al. 2014).

The mismatch repair (MMR) system is a critical DNA repair pathway for recognizing and repairing DNA base mismatches, insertions and deletions that arise during DNA replication (Liu et al. 2017) or promoting apoptosis if DNA damage is severe (Hassen et al. 2016). In different cancer types, a mutation within the tumor cascade causes a deficiency in the MMR system (MMRd), resulting in a genomic instability of the microsatellites (Yamamoto and Imai 2015). Thus, MMRd and high microsatellite instability (MSI-H) are closely related (Pai and Pai 2016; Smyth et al. 2017; Svrcek et al. 2019).

Roughly 8–9% (Smyth et al. 2017; Polom et al. 2018; Pietrantonio et al. 2019) of GC are MSI-H. In a whole-exome data analysis of 11,139 tumor-normal pairs, GC was the third most common cancer type with MSI-H (Bonneville et al. 2017). Most MSI-H/ MMRd GC are sporadic and less than 2% of patients with Lynch syndrome (carriers of hereditary MMR mutations) are diagnosed with GC (Capelle et al. 2010). It has been shown that MSI-H/ MMRd is a positive prognostic biomarker in GC (Fang et al. 2012; Marrelli et al. 2016; Zhang et al. 2018; Polom et al. 2018; Kohlruss et al. 2019; Pietrantonio et al. 2019). In practice GC is not routinely tested for MSI/ MMR status, unless individual patients show indicators of Lynch syndrome (Smyth et al. 2016; Moehler et al. 2019).

For patients with a locally advanced resectable GC, perioperative or postoperative fluoropyrimidine/platinum based chemotherapy confer a survival benefit (Paoletti et al. 2010; Smyth et al. 2016; Moehler et al. 2019). The cytostatic effect of fluoropyrimidines is based on the incorporation into the DNA during replication and the alteration of the nucleotide precursor pool (Li et al. 2016). Platinum-based drugs are cross-linking the DNA molecules (Hato et al. 2014). The resulting DNA damages activate the MMR system, which in turn induces apoptosis (Dasari and Bernard Tchounwou 2014). However, this mechanism may be attenuated in the subgroup of patients with MMRd GC.

A post hoc analysis of the British MAGIC trial found MMRd as a positive predictor of overall survival in patients treated with surgery only and as a negative predictor of overall survival when treated with an additional perioperative ECF chemotherapy for resectable GC (Cunningham et al. 2006; Smyth et al. 2017). A retrospective study from South Korea including 881 patients with stage II and III GC suggests that those with MMRd tumors do not benefit from adjuvant chemoradiotherapy with 5-fluorouracil/leucovorin regarding disease-free survival (DFS) (Kim et al. 2020). Another retrospective study from Japan including 285 GC patients showed that loss of the MMR-protein MLH1 was associated with chemoresistance and did not prolong recurrence-free survival of GC patients following neoadjuvant S−1/platinum-based chemotherapy (Hashimoto et al. 2019).

The impact of MSI on survival was analysed in a meta-analysis of four prospective trials that investigated the role of perioperative (MAGIC trial) or postoperative chemotherapy (CLASSIC, ARTIST and ITACA-S trials) for patients with resectable GC. In the meta-analysis, patients with MSI-H GC did not benefit from additional chemotherapy (Pietrantonio et al. 2019). However, besides the MAGIC trial, no other trials have investigated the role of perioperative chemotherapy for resectable MSI-H/ MMRd GC so far.

The objective of this study was to investigate the prognostic impact of therapy with surgery alone compared to additional perioperative chemotherapy on overall survival (OS) of GC patients depending on their MMR status in a retrospective analysis and a meta-analysis.

## Methods

### Study population

We selected our study population from the staR (Gastric Cancer Research) project, a database of patients with current or past diagnosis of GC, including cancers of the esophagogastric junction Siewert type II and III, excluding GC other than adenocarcinoma. A total of 816 patients were recruited between April 2013 and December 2018 by the German centers of the staR project. Clinical data include vital record data, date of initial diagnosis, TNM category, Lauren-type, site of the GC, whether surgery was performed on the cancer and whether a chemotherapy was administered and which type (neoadjuvant, adjuvant, perioperative, palliative).

**Fig. 1:**
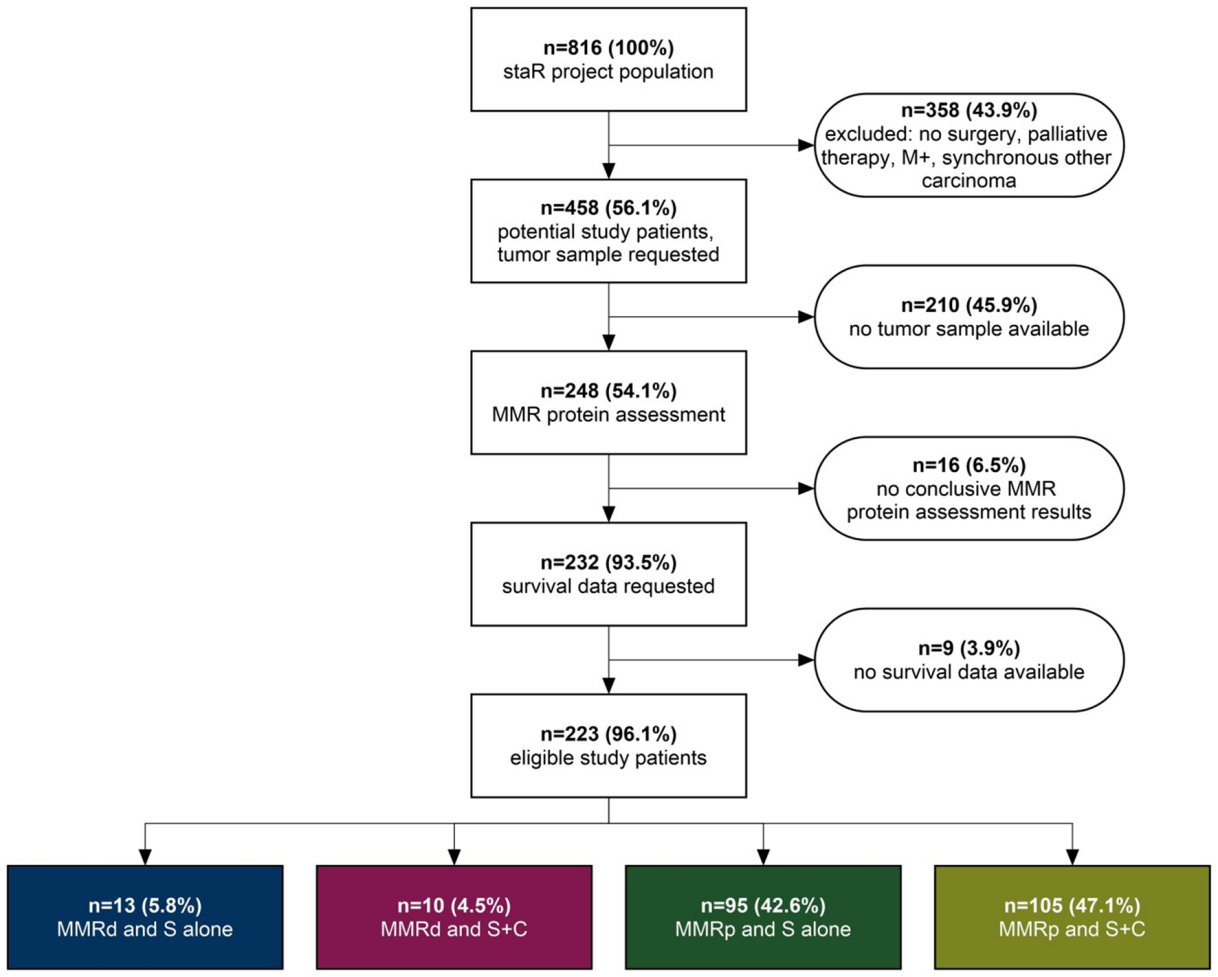
Flowchart of the enrolment of study patients. *M + * metastases, *S alone* surgery alone, *S + C* surgery plus Chemotherapy, *MMRd* mismatch repair deficient, *MMRp* mismatch repair proficient

Figure 1 shows the recruitment of study patients. For our study, we selected all patients from the staR database who underwent GC surgery and had either no chemotherapy (S only) or perioperative chemotherapy (S + C) administered. Exclusion criteria were metastases (M +), a not curatively intended therapy and the occurrence of synchronous other carcinoma. A number of 458 patients remained.

For those 458 patients, we contacted the respective (86 individual) Institutes of pathology that reviewed of the tissue samples obtained by the surgical intervention (in exceptions: the biopsy obtained via gastroscopy at the date of initial diagnosis). The samples were requested as a loan to perform the MMR protein assessment. Tumor samples were not available for 210 patients.

### MMR protein assessment

MMR protein assessment was performed on cancer tissues of 248 patients. The samples were prepared in 2 µm sections and stained for MLH1, MSH2, MSH6 and PMS2 using Ventana anti-MLH1 and anti-PMS2 mouse monoclonal antibodies (Ventana) and MSH2 and MSH6 mouse monoclonal antibody (CellMarque) on the VENTANA BenchMark ULTRA instrument.

The signals are classified as intact or loss based on nuclear localization only. A tumor section was designated a loss of MMR protein expression when the malignant epithelial cells had no nuclear staining, whilst nuclei of lymphocytes and stromal cells or normal, non-neoplastic epithelial cells were stained positive in vicinity of the tumor. Unequivocal nuclear staining of any intensity above background by cancer cells was considered sufficient MMR protein expression.

Detection of all four proteins in the tumor indicated proficient mismatch repair status (MMRp). The sample was considered MMRd if at least one of the proteins (MLH1, MSH2, MSH6 or PMS2) was lost.

### Survival data

To obtain current survival data, we contacted the patients themselves, their general practitioner or oncologist or the respective cancer registry for 232 patients. The last date of follow-up was in June 2020. No survival data were determinable for nine patients. OS was calculated from the date of initial diagnosis.

### Statistical analysis and matching

The final 223 GC patients were divided into subgroups, regarding chemotherapy status and MMR-protein status. The four subgroups are: MMRd and S only (*n* = 13), MMRd and S + C (*n* = 10), MMRp and S only (*n* = 95), MMRp and S + C (*n* = 105). The subgroups were compared pairwise for baseline clinical characteristics gender, age, T and N category, cancer site and Lauren type using Microsoft Excel (Version 14.0) and an online two-sided Fisher’s exact test (Motulsky 2021). *P* < 0.05 was considered statistically significant.

The two MMRp subgroups showed statistically significant differences in clinical characteristics (T and N category). They were matched in an 1:1 fashion for gender (female and male), age at date of initial diagnosis (difference max. ± 5 years, in exceptions ± 10 years), T category (pT1, pT2, pT3 and pT4, in exceptions pT1–2 and pT3–4) and N category (pN0 and pN +). A total number of 98 matched patients with MMRp GC remained.

For the survival analysis, the sets of unmatched data of the MMRd subgroups S only vs. S + C and the matched data of the MMRp subgroups S only vs. S + C were used. Kaplan–Meier survival analysis and Cox-regression were carried out using SPSS Statistics (Version 28.0). Median OS was not calculated as it was not reached.

### Systematic review

The Medline-Database (PubMed) was drafted for eligible studies employing the following search terms: ‘‘gastric cancer’’ AND ‘‘chemotherapy’’ AND ‘‘mismatch repair’’ (all fields), with an end-date of 2nd February 2021. The Boolean operator ‘‘AND’’ was used to narrow the search results, returning 73 search results.

For inclusion in the meta-analysis, a study had to meet the following criteria: participants diagnosed with gastric carcinoma and surgical removal of the tumor, including stratification into subgroups regarding MMR-protein status and subgroups with or without neoadjuvant or perioperative chemotherapy, assessed for OS. Papers were excluded if they did not include outcome data. Identified papers were initially screened by title and abstract for not fulfilling the inclusion criteria and then reviewed in detail for meeting the inclusion criteria. One study was eligible for the meta-analysis (Smyth et al. 2017).

The risk of bias of the study was assessed for collection of the clinical data, histopathological methods, survival analysis, selective or incomplete outcome reporting and for-profit bias. Data were extracted from the published outcomes.

### Meta-analysis

We performed a meta-analysis using two sets of data, the survival data regarding MMR status published by Smyth et al. (comprising a Kaplan–Meier survival plot and number at risk in 1-year intervals) (Smyth et al. 2017) and the individual patient data of MMRd cases and matched MMRp cases of our study.

Survival data at given times were extracted from the Kaplan–Meier plot using DigitizeIt (Version 2.5). As the total number of patients within the MMRd subgroups was low (*n* = 21), the number of events and number at risk could be estimated in 6-month time intervals. For the larger number of MMRp patients (*n* = 243) the number of events was estimated in 1-year intervals using the published number at risk and the method by Tierney et al. (2007). This provided a chart of estimated number of events and number at risk in 6-month/ 1-year time intervals for each subgroup. For the MMRd and MMRp subgroups ,hazard ratios (HR) were calculated using an online Hazard Ratio Calculator (Georgiev 2021).

Survival data of the MMRd and MMRp subgroups from the two studies were joined in meta-analyses using Stata/MP (Version 17.0). For HR, *P* < 0.05 was considered statistically significant. To assess variation across the studies, statistical evaluation of heterogeneity by Cochran’s *Q* was used and heterogeneity was considered to be present if Cochran’s *Q* delivered *P* < 0.05. An *I*^2^ statistic was used to quantify the proportion of variation in the treatment effect in the study that is due to heterogeneity rather than chance.

## Results

### Clinical and histopathological characteristics

Table 1 shows clinical characteristics amongst the four study subgroups, including the median and range of age at initial diagnosis, the distributions of gender, T and N category, cancer site and Lauren type, and respective *P* values. Data on MMR status and survival were available for a total of 223 patients. 23 patients (10.3%) had MMRd GC.

**Table 1:** Clinical characteristics of the unmatched study subgroups

	Total (%)	MMRd and S alone (%)	MMRd and S + C (%)	*P* value MMRd	MMRp and S alone (%)	MMRp and S + C (%)	*P* value MMRp
**n**	223 (100.0)	13	10		95	105	
*Gender*							
Female	83 (37.2)	6 (46.2)	3 (30.0)	0.67	32 (33.7)	42 (40.0)	0.38
Male	140 (62.8)	7 (53.8)	7 (70.0)		63 (66.3)	63 (60.0)	
**Age**							
Median (yrs.)	66	74	65		69	63	
Range (yrs.)	31–89	50–89	46–75		31–87	31–80	
**T category**							
pT1	80 (35.9)	5 (38.5)	3 (30.0)		53 (55.8)	19 (18.1)	
pT2	42 (18.8)	3 (23.1)	4 (40.0)		15 (15.8)	20 (19.0)	
pT3	69 (30.7)	4 (26.7)	2 (20.0)		17 (17.9)	46 (43.8)	
pT4	25 (11.1)	0 (0.0)	1 (10.0)		7 (7.4)	17 (16.2)	
pT1–2	122 (54.7)	8 (61.5)	7 (70.0)	1.00	68 (71.6)	39 (37.1)	0.0001***
pT3–4	94 (41.8)	4 (26.7)	3 (30.0)		24 (25.3)	63 (60.0)	
T missing	7	1	0		3	3	
**N category**							
pN0	122 (54.7)	11 (84.6)	6 (60.0)	0.34	63 (66.3)	42 (40.0)	0.0001***
pN +	90 (0.4)	2 (15.4)	4 (40.0)		25 (26.3)	59 (56.2)	
N missing	11	0	0		7	4	
**Site**							
Cardia	47 (20.9)	0 (0.0)	4 (40.0)	0.04*	11 (11.6)	32 (30.5)	0.0008***
Non-cardia	163 (73.1)	11 (84.6)	6 (60.0)		81 (85.3)	65 (61.9)	
Site missing	13	2	0		3	8	
**Lauren type**							
Intestinal	128 (56.9)	10 (76.9)	8 (80.0)	1.00	56 (58.9)	54 (51.4)	0.23
Diffuse	75 (33.6)	1 (7.7)	1 (10.0)		30 (31.6)	43 (41.0)	
Mixed	16 (7.2)	1 (7.7)	0 (0.0)		8 (8.4)	7 (6.7)	
Missing	4	1	1		1	1	

Comparing the subgroups pairwise, we found statistically significant differences between the two MMRp subgroups regarding T category (*p* < 0.001; 39 pT1-2 and 63 pT3–4 in S + C vs. 68 pT1-2 and 24 pT3–4 in S only), N category (*p* < 0.001; 42 pN0 and 59 pN + in S + C vs. 63 pN0 and 25 pN + in S only) and cancer site (*p* < 0.001; 32 cardia and 65 non-cardia in S + C vs. 11 cardia and 81 non-cardia in S only) and between the two MMRd subgroups regarding cancer site (*p* = 0.035; 4 cardia and 6 non-cardia in S + C vs. 0 cardia and 11 non-cardia in S only).

Table 2 shows the clinical characteristics of the MMRp subgroups after matching in the mentioned manner.

**Table 2:** Clinical characteristics of the matched MMRp subgroups

	Total (%)	MMRp and S alone (%)	MMRp and S + C (%)	*P* value
**n**	98 (100.0)	49	49	
*Gender*				
Female	40 (40.8)	20 (40.8)	20 (40.8)	1.00
Male	48 (49.0)	29 (59.2)	29 (59.2)	
**Age**				
Median (yrs.)	70	70	68	
Range (yrs.)	44–87	44–87	46–80	
**T category**				
pT1	39 (39.8)	21 (42.9)	18 (36.7)	0.59
pT2	21 (21.4)	9 (18.4)	12 (24.5)	
pT3	27 (27.6)	13 (26.5)	14 (28.6)	1.00
pT4	11 (11.2)	6 (12.2)	5 (10.2)	
pT1–2	60 (63.2)	30 (61.2)	30 (61.2)	1.00
pT3–4	28 (28.6)	19 (38.8)	19 (38.8)	
T missing	0	0	0	
**N category**				
pN0	54 (55.1)	27 (55.1)	27 (55.1)	1.00
pN +	44 (44.9)	22 (44.9)	22 (44.9)	
N missing	0	0	0	

Of the ten patients with MMRd GC who were treated with perioperative chemotherapy three received doublet (5-fluoruracile plus cis- or oxaliplatin), five received triplet (additional docetaxel or eiprubicine) and two received an unknown chemotherapy regimen. Of the 49 matched patients with MMRp GC who were treated with perioperative chemotherapy 9 received doublet, 34 received triplet and 6 received an unknown chemotherapy regimen.

Of the 23 samples with MMRd GC, a number of 20 (87%) showed classical pairwise loss of MMR protein expression in either only MLH1 and PMS2 (*n* = 17) or only MSH2 and MSH6 (*n* = 3).

### Survival

**Fig. 2:**
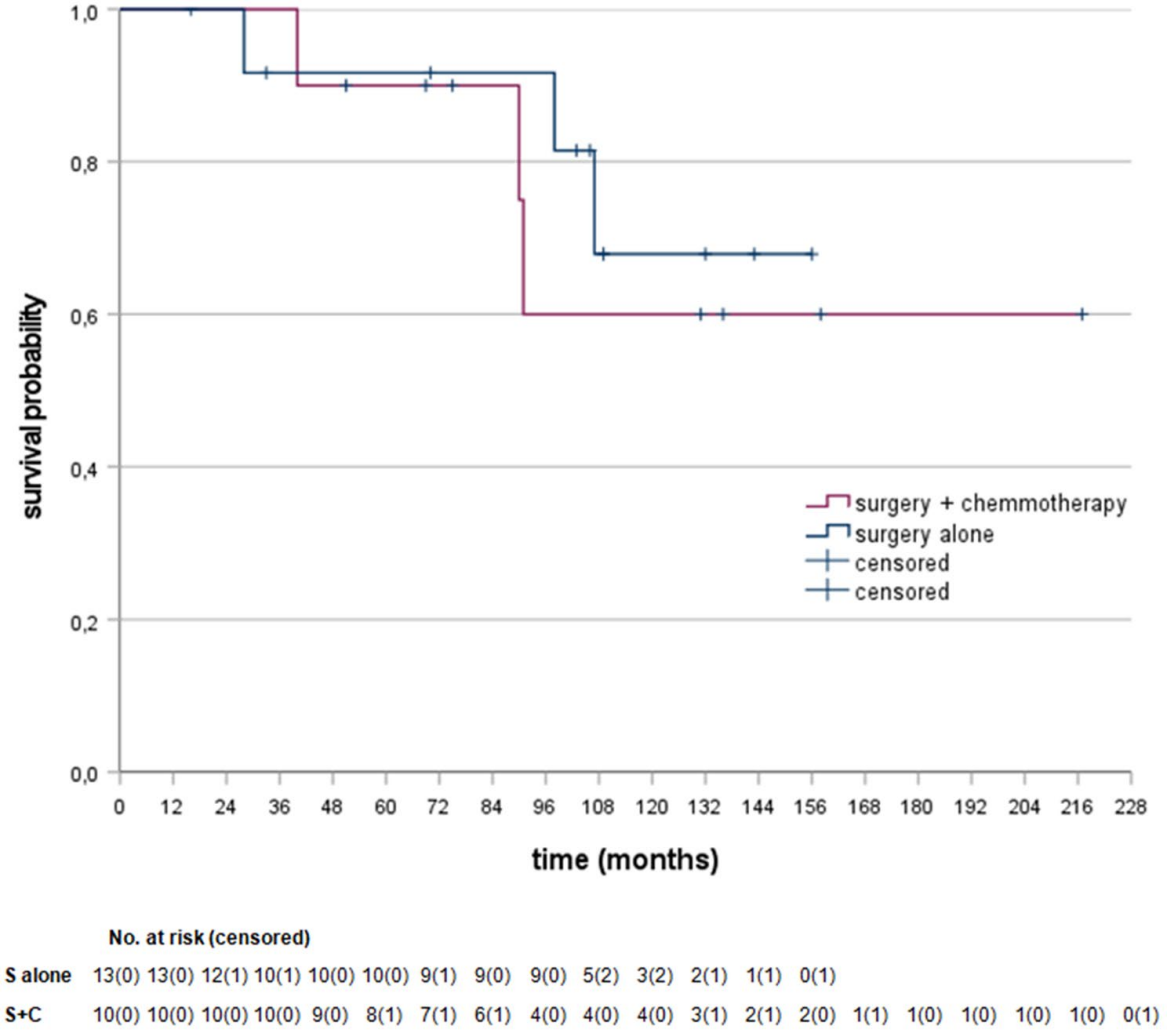
Kaplan–Meier curves for the MMRd subgroups. S alone vs. S + C stratified hazard ratio for death, HR = 0.67 (95% CI 0.13–3.37, *P* = 0.63), No. of deaths: 3/13 (23.1%) in S alone, 3/10 (30.0%) in S + C

**Fig. 3:**
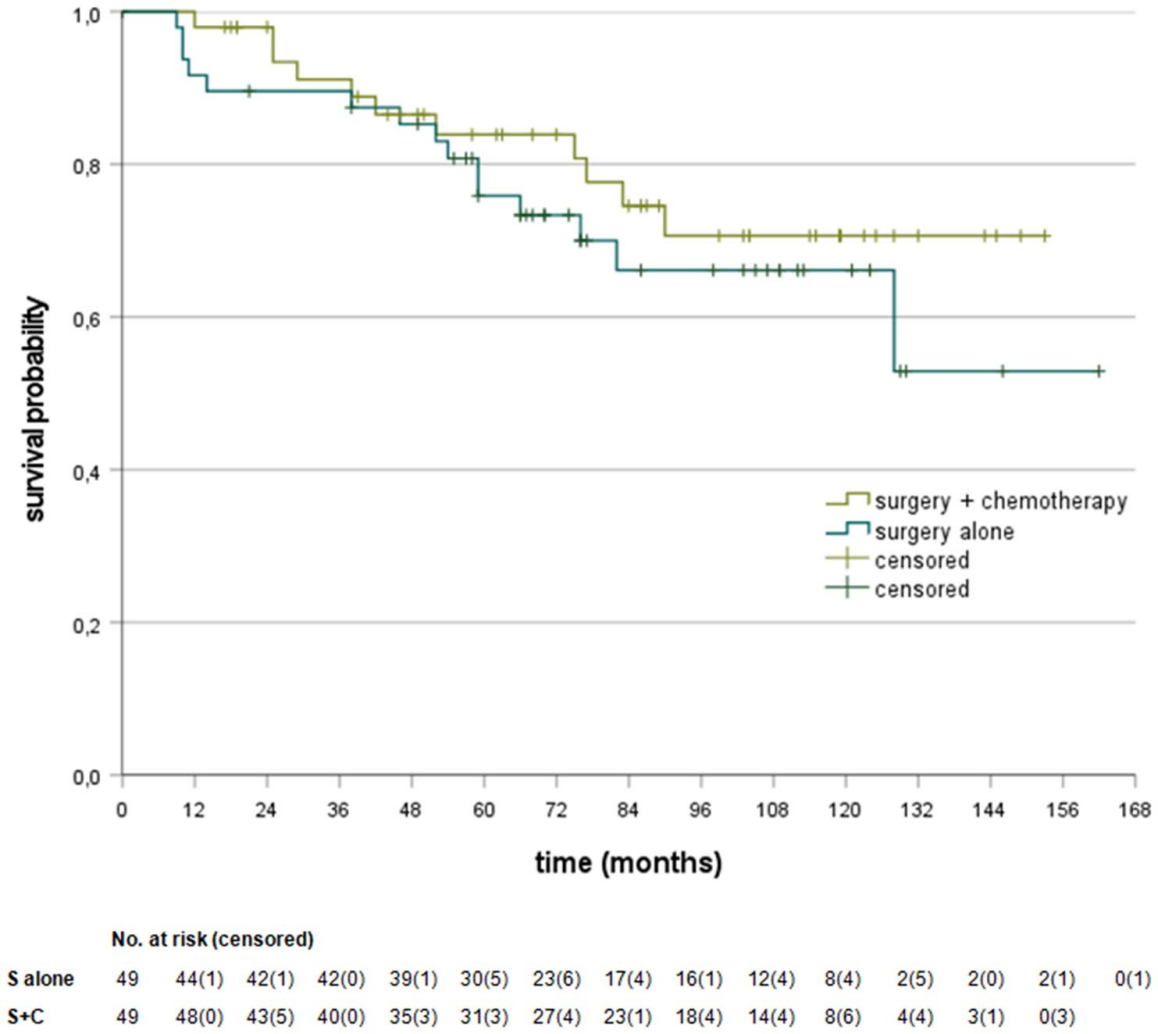
Kaplan–Meier curves for the matched MMRp subgroups. S alone vs. S + C stratified hazard ratio for death, HR = 1.44 (95% CI 0.66–3.13, *P* = 0.36), No. of deaths: 15/49 (30.6%) in S alone, 11/49 (22.4%) in S + C

Figures 2 and 3 show the Kaplan–Meier survival curves of the MMRd and MMRp subgroups. Comparing surgery alone to perioperative chemotherapy HR was 0.67 (95% CI 0.13–3.37, *P* = 0.63) for patients with MMRd GC. Comparing surgery alone to perioperative chemotherapy HR was 1.44 (95% CI 0.66–3.13, *P* = 0.36) for the matched patients with MMRp GC. No statistically significant differences in OS were found amongst any of these groups. Median OS was not reached in any of the subgroups.

### Meta-analysis

Estimating the survival data from the published data by Smyth et al. (2017) shows for the MMRd subgroups a HR of 0.26 (95% CI 0.08–0.82, *P* = 0.03) comparing surgery alone to perioperative chemotherapy, and for the MMRp subgroups a HR of 1.14 (95% CI 0.84–1.57, *P* = 0.39) comparing surgery alone to perioperative chemotherapy.

**Fig. 4:**
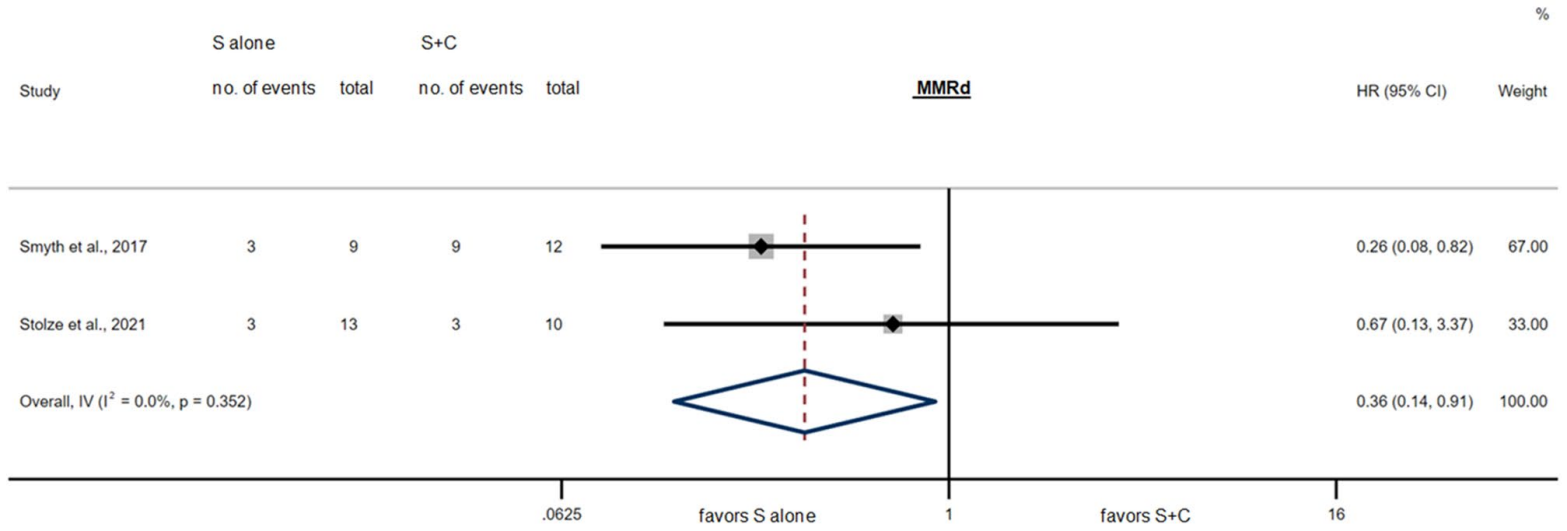
Survival for S alone vs. S + C in the MMRd subgroups for individual studies and in meta-analysis, HR = 0.36 (95% CI 0.14–0.91, *P* = 0.03*), Cochran’s *Q* = 0.87 (*P* = *0.35*), *I*^2^ = 0.0%

**Fig. 5:**
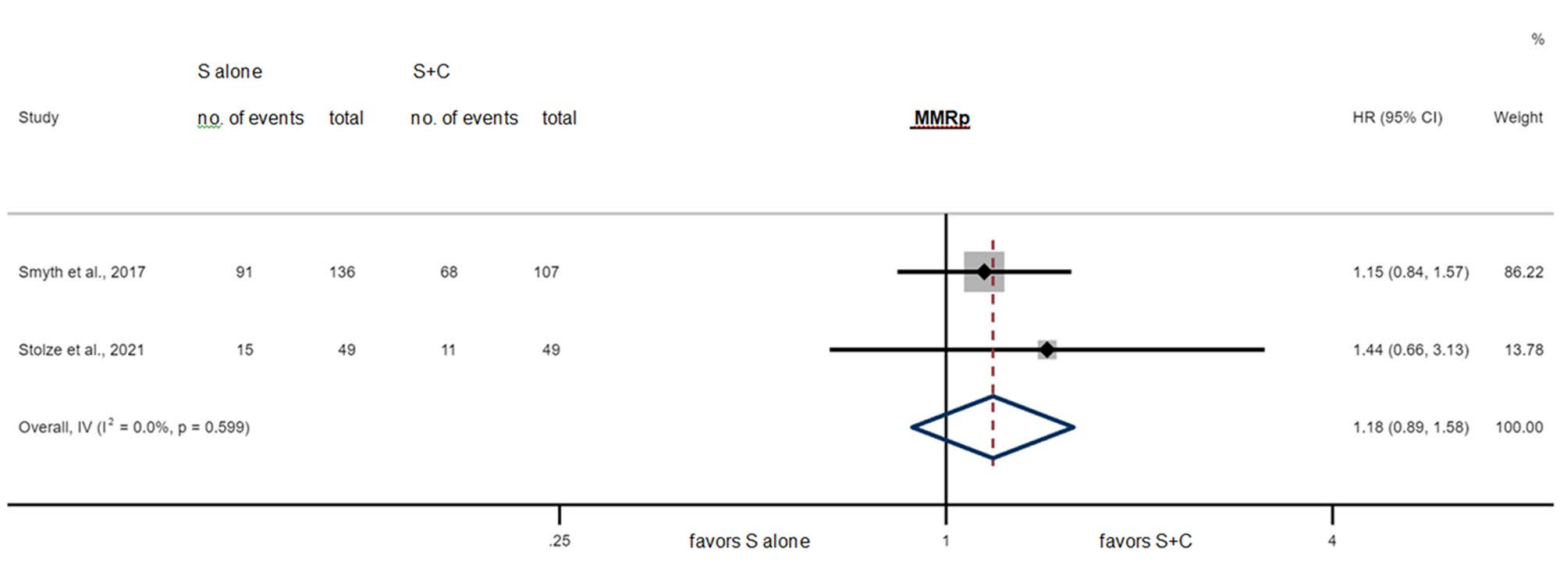
Survival for S alone vs. S + C in the MMRp subgroups for individual studies and in meta-analysis, HR = 1.18 (95% CI 0.89–1.58, *P* = 0.26), Cochran’s *Q* = 0.28 (*P* = 0.60), *I*^2^ = 0.0%

Figures 4 and 5 show the results of the meta-analysis. It included 341 MMRp patients showing a HR of 1.18 (95% CI 0.89–1.58, *P* = 0.26) comparing surgery alone to perioperative chemotherapy and 44 MMRd patients showing a significantly better OS with a HR of 0.36 (95% CI 0.14–0.91, *P* = 0.03) when treated with surgery alone compared to perioperative chemotherapy.

Both subgroups showed high degrees of similarity/ non-heterogeneity (Cochran’s *Q* = 0.87 for MMRd, Cochran’s *Q* = 0.28 for MMRp, *I*^2^ = 0.0% in either case).

## Discussion

In our study, GC patients treated by surgery alone who had MMRd showed improved OS compared to those who received surgery plus chemotherapy. On the contrary, GC patients who had MMRp, showed an impaired OS if treated with surgery alone, compared to surgery plus chemotherapy. Our meta-analysis further emphasizes these results and strengthens the finding that in GC patients who have MMRd, a fluoropyrimidine/platinum based perioperative chemotherapy may be futile or even detrimental and may be omitted.

In our meta-analysis, the advantage of perioperative chemotherapy over surgery only, could not be shown in MMRp patients (*n* = 341). Likely, the number of patients included in our meta-analysis and thus the statistical power were insufficient compared to those of the MAGIC (*n* = 503) trial, where the efficacy of perioperative chemotherapy in GC was shown.

The small number of patients with resectable MMRd GC included in our study and meta-analysis does not allow stratification for N category (Smyth et al. 2017; Pietrantonio et al. 2019). However, in patients with resectable GC, preoperative N category is not reliable and thus not contemplated for clinical decision-making (Smyth et al. 2016; Moehler et al. 2019). Differently, in patients with MSI-H CRC, the N category determined postoperatively is reliable and crucial to select patients for adjuvant chemotherapy (Argilés et al. 2020; Cohen et al. 2021).

The better survival prognosis of patients with MSI-H/MMRd GC has been linked to a strong lymphocytic infiltration observed particularly in locally advanced, radically resected tumors, as it might attenuate the risk of developing micrometastases after surgery (Grogg et al. 2003; Chiaravalli et al. 2006; Giampieri et al. 2017). In MMRd cancers, tumor-infiltrating lymphocytes show an elevated expression of PD-1, a mechanism that suppresses anti-tumor immune response (van Velzen et al. 2020). Checkpoint inhibitor therapy targeting PD-1/PDL-1 restore immune system function and represents the current standard of care for patients with metastatic MSI-H/MMRd CRC (André et al. 2020) and represent a therapy option for metastatic MMRd GC (Mishima et al. 2019; Marabelle et al. 2020; Kubota et al. 2020). Furthermore, two phase-II trials are currently investigating the role of perioperative checkpoint blockade for patients with MMRd GC (Jabbour 2017; Cohen et al. 2020).

Our meta-analysis and a previous meta-analysis show that MMRd and MSI-H are good candidate biomarkers to select GC patients for perioperative chemotherapy, respectively (Pietrantonio et al. 2019). In a clinical setting both MMR protein assessment by immunohistochemistry (IHC) and MSI testing by polymerase chain reaction (PCR) are equally valid in detecting MSI-H/MMRd in tumor samples (Pai and Pai 2016; Smyth et al. 2017; Svrcek et al. 2019). Generally, MMR protein assessment is less standardized, less reproducible and requires a more experienced pathologist, but is also less cost- and time-consuming compared to MSI testing (Svrcek et al. 2019).

## Limitations

MMR protein assessment was performed retrospectively on existing tumor samples. The MMR status could be assessed on tumor specimens of 248 out of 458 initially identified patients. Indeed, many Institutes of pathology have limited personnel resources that prevent them to ship the histological specimens. Additionally, the low prevalence of MMRd prevented subgroup analyses, in particular according to the lymph nodal status. Furthermore, sufficient reliable data on DFS were difficult to obtain because of the retrospective study design in conjunction with the Germany-wide distribution of staR-project patients.

For the meta-analysis, we retrieved only one further publication (Smyth et al. 2017) reporting on MMR protein status, surgery with or without perioperative chemotherapy and survival of GC patients. Unfortunately, clinical characteristics of the MMRd and MMRp subgroups were not available. Survival data were estimated from the published Kaplan–Meier plot and number at risk, which may limit the accuracy of the collected data. However, we strictly adhered to the rules for data estimation to overcome this limitation (Tierney et al. 2007). Compared to the MAGIC trial, the fluoropyrimidine/platinum-based chemotherapy regimens used in our cohort were heterogeneous.

## Conclusions

Our study further strengthens the concept that the subgroup of patients with MMRd GC may not benefit from standard perioperative chemotherapy. Thus, the MMR status is a candidate predictive biomarker in curatively resectable GC, avoiding unnecessary treatment for patients with MMRd GC. Our meta-analysis currently represents the best evidence regarding the futile effect of standard perioperative chemotherapy on patients with MMRd GC. However, a prospective trial with an appropriate number of participants is urgently needed, to clarify whether the subgroup of patients with MMRd GC and histologically confirmed local lymph node metastases may still have a survival benefit from adjuvant chemotherapy, as observed in patients with MMRd CRC (Cohen et al. 2021). Prospective trials investigating the role of checkpoint inhibitors for patients with locally advanced MSI-H/MMRd GC are ongoing (Jabbour 2017; Mishima et al. 2019; Marabelle et al. 2020; Cohen et al. 2020; Kubota et al. 2020).
